# Genetic variations in the *DYNC2H1* gene causing SRTD3 (short-rib thoracic dysplasia 3 with or without polydactyly)

**DOI:** 10.3389/fgene.2023.1125473

**Published:** 2023-04-06

**Authors:** Wenqi Chen, Yazhou Li, Jing Zhang, Yufan Yuan, Donglan Sun, Jiayu Yuan, Kai Yang, Ying Liang, Qing Guo

**Affiliations:** ^1^ Prenatal Diagnosis Center, Shijiazhuang Obstetrics and Gynecology Hospital, Key Laboratory of Maternal and Fetal Medicine of Hebei Province, Shijiazhuang, Hebei, China; ^2^ Department of Pediatric Orthopaedic, The Third Hospital of Hebei Medical University, Shijiazhuang, Hebei, China; ^3^ Prenatal Diagnosis Center, Beijing Obstetrics and Gynecology Hospital, Beijing Maternal and Child Healthcare Hospital, Capital Medical University, Beijing, China; ^4^ Reproductive Medicine Center, Shijiazhuang Obstetrics and Gynecology Hospital, Shijiazhuang, Hebei, China

**Keywords:** *DYNC2H1* gene, short-rib thoracic dysplasia 3 (SRTD3), skeletal dysplasia, whole-exome sequencing, prenatal diagnosis

## Abstract

**Background and aims:** Short-rib thoracic dysplasia 3 with or without polydactyly (SRTD3) represents a type of severe fetal skeletal dysplasia (SD) characterized by shortened limbs, narrow thorax with or without polydactyly, which is caused by the homozygous or compound heterozygous mutations in the *DYNC2H1* gene. SRTD3 is a recessive disorder, identification of the responsible genetic variation would be beneficial to an accurate prenatal diagnosis and well-grounded counseling for the affected families.

**Material and methods:** Two families having experienced recurrent fetal SDs were recruited and submitted to a multiplatform genetic investigation. Whole-exome sequencing (WES) was performed with samples collected from the probands. Sanger sequencing and fluorescent quantitative PCR (qPCR) were conducted as validation assays for suspected variations.

**Results:** WES identified two compound heterozygous variations in the *DYNC*2H1(NM_001080463.2) gene, namely c.2386C>T (p.Arg796Trp) and c.7289T>C (p.Ile2430Thr) for one; and exon (64–83)del and c.8190G>T (p.Leu2730Phe) for the other, respectively. One variant in them, exon (64–83)del, was novelly identified.

**Conclusion:** The study detected two compound heterozygous variation in *DYNC2H1* including one novel deletion: exon (64–83) del. Our findings clarified the cause of fetal skeletal dysplasia in the subject families, provided guidance for their future pregnancies, and highlighted the value of WES in diagnosis of skeletal dysplasia with unclear prenatal indications.

## Introduction

Short-rib thoracic dysplasia (SRTD) with or without polydactyly is an umbrella term of a group of genetically heterogeneous skeletal dysplasias consistent with the autosomal recessive (AR) inheritance pattern. SRTDs are characterized by short ribs, short limbs, constricted thoracic cage, a ‘trident’ appearance of the acetabular roof and anomalies in kidney, heart, liver, pancreas, genitalia and intestine (Chen and Tzen, 2001; Chen et al., 2002; Chen et al., 2003; Chen et al., 2005; Chen et al., 2012a; Chen et al., 2012b). Currently, SRTD are classified into the categories of short-rib thoracic dysplasia with or without polydactyly type 1–21 (SRTD1-21) (Table 1).

**TABLE 1:** SRTD are classified into the categories of SRTD1-21.

Phenotype	Location	PhenotypeMIM number	Gene	Gene MIM number	Inheritance
SRTD1	15q13	208500	SRTD1	208500	AR
SRTD2	3q25.33	611263	IFT80	611177	AR
SRTD3	11q22.3	613091	DYNC2H1	603297	AR,DR
SRTD4	2q24.3	613819	TTC21B	612014	AR
SRTD5	4p14	614376	WDR19	608151	AR
SRTD6	4q33	263520	NEK1	604588	AR,DR
SRTD7	2p24.1	614091	WDR35	613602	AR
SRTD8	7q36.3	615503	WDR60	615462	AR
SRTD9	16p13.3	(266920)	IFT140	614620	AR
SRTD10	2p23.3	615630	IFT172	607386	AR
SRTD11	9q34.11	615633	WDR34	613363	AR
SRTD12	not mapped	269860	SRTD12	269860	AR
SRTD13	5q23.2	616300	CEP120	613446	AR
SRTD14	14q23.1	616546	KIAA0586	610178	AR
SRTD15	2p21	617088	DYNC2LI1	617083	AR
SRTD16	20q13.12	617102	IFT52	617094	AR
SRTD17	3q29	617405	TCTEX1D2	617353	AR
SRTD18	14q24.3	617866	IFT43	614068	AR
SRTD19	12q24.11	617895	IFT81	605489	AR
SRTD20	4q28.1	617925	INTU	610621	AR
SRTD21	17p13.1	619479	KIAA0753	617112	AR

The short-rib thoracic dysplasia 3 with or without polydactyly (SRTD3; OMIM #613091) refers to a rare subtype of SRTDs, characterized by the constricted thoracic cage, shortened limbs, and associated visceral abnormalities with or without polydactyly. SRTD3 belongs to the “ciliopathies with major skeletal involvement” conditions according to the revised consensus workshop (Bonafe et al., 2015), and is caused by homozygous or compound heterozygous mutations in the *DYNC2H1* gene (dynein, cytoplasmic 2, heavy chain 1; OMIM #603297), which encodes a component of the cytoplasmic dynein complex (Dagoneau et al., 2009; McInerney-Leo et al., 2015). This complex is associated with the ciliary intraflagellar transport (IFT), an evolutionarily conserved process that is necessary for ciliogenesis and plays an important role in Hedgehog (Hh), Wnt, Platelet-derived growth factor (PDGF), Notch, G-Protein coupled receptor (GPCR), Mammalian target of rapamycin (mTOR), Transforming growth factor beta (TGF-β) and Calcium signaling pathways (Schmidts et al., 2013a; Schmidt et al., 2015).

In general, SRTD3 is lethal in the neonatal period due to respiratory insufficiency secondary to the severely restricted thoracic cage, whereas other SRTD subtypes are compatible with life (Huber and Cormier-Daire, 2012; Schmidts et al., 2013b). Although prenatal ultrasonography could detect the skeletal abnormalities of SRTD3, this condition was often difficult to be precisely diagnosed before birth. Since SRTD3 is a recessive disorder, identification of the responsible genetic variation would be beneficial to an accurate prenatal diagnosis and well-grounded counseling for the affected families.

In the present study, two families with experiences of multiple adverse gestations including recurrent fetal skeletal dysplasias were recruited. Prenatal ultrasonography examination and genetic detection were conducted to identify the causes of these manifestations in affected fetuses.

## Materials and methods

### Subjects

Two unrelated cases were recruited between March 2020 and April 2021 at the Prenatal Diagnosis Center, Shijiazhuang Obstetrics and Gynecology Hospital. A comprehensive prenatal ultrasonic examination was conducted on the patients. We carried out a thorough clinical survey. Subsequently, the peripheral blood samples of trio family members in the two pedigrees were collected for the following genetic detection. The studies involving human participants were reviewed and approved by The Ethics Committee of Shijiazhuang Obstetrics and Gynecology Hospital.

### Genomic DNA extraction

Amniocentesis was performed to obtain the fetal cell samples, along with 3 ml of peripheral blood collected from the parents using BD Vacutainer™ tubes (BD Biosciences, New Jersey, United States). Genomic DNA was extracted with the QIAamp DNA Blood Mini-Kit (Qiagen Sciences, New York, United States), and the DNA quality was validated by 1% agarose gels and the Qubit^®^ 2.0 Flurometer (Life Technologies, CA, United States).

### Whole-exome sequencing

WES was performed by MyGenomics, Inc. (Changping, Beijing, China) as described in our previous study (Zhang et al., 2021). Briefly, the enrichment of the exonic region sequences was conducted by the Sure Select Human Exon Sequence Capture Kit (Agilent, United States). The sequencing library was quantified using the Illumina DNA Standards and Primer Premix Kit (Kapa Biosystems, United States), and was massively parallel-sequenced using the Illumina Novaseq6000 platform. After sequencing and filtering out the low-quality reads, the high-quality data (with general quality level Q30 reads >89%) was aligned to the human genome reference sequence [hg19] using Burrows-Wheeler Aligner tool. The third-party software GATK (https://softw are.broad institute.org/gatk/) and the Verita Trekker^®^ Variants Detection system (Berry Genomics, China) were employed for variant calling. Variants with lower quality (read depth<10x, allele fraction<30%) were eliminated. The variations were identified by sequence alignment with the NCBI Reference Sequence (NG_016423.2, NP_001073932.1, NM_001080463.2) using Chromas v2.33. The pathogenicity of the identified variants was then assessed according to the common guidelines issued by the American Association of Medical Genetics and Genomics (ACMG) (Richards et al., 2015) referring to multiple databases (1000g2015aug_eas, https://www.internationalgenome.org/; ExAC_EAS, http://exac.broadinstitute.org; gnomAD_exome_EAS, http://gnomad.broadinstitute.org/; HGMD^®^: Human Gene Mutation Database Professional v.2021.10) with the Enliven^®^ Variants Annotation Interpretation (Berry Genomics, China) system.

### Validation experiments

The suspected diagnostic variants were validated by Sanger sequencing using ABI 3730 Automated Sequencer (Applied Biosystems, United States) according to the manufacturer’s protocol. Fluorescent quantitative PCR (qPCR) was also carried out to verify the suspected deletion variant identified in Case 2.

### Analysis of missense variants

The evolutionary conservatism of amino acid (AA) residues affected by specific missense variants was analyzed using UGENE (http://ugene.net/) with default parameters.

### Structural analysis

Referring to the crystal structure of 6rla. 1. A (Toropova et al., 2019) protein, the DYNC2H1 protein structures at 2241–2520 and 2611–2880 regions were constructed by Swiss-model program. The Swiss-Pdb Viewer program was referred to modeling the wild-type (WT) and DYNC2H1: p. Ile2430Thr and p. Leu2730Phe mutant models of DYNC2H1 protein segments. The molecular dynamics (MD) prediction analysis was generated by GROMACS (version 2020.6) (Rakhshani et al., 2019). We carried out 60 ns MD simulations on the DYNC2H1-WT, DYNC2H1-Ile2430Thr, and DYNC2H1-Leu2730Phe models. The CHARMM36 force field was applied to add hydrogen atoms and N-terminal and C-terminal patches to the models (Soteras Gutiérrez et al., 2016). The wild type or the mutant structure of the protein was immersed in cubic boxes which contains water and placed at least 1.0 nm from the box edge. Na^+^ and Cl^−^ ions were used for neutralization. The MD simulations were performed at a temperature of 300K for 60 ns after energy minimization, equilibration. The following GROMACS distribution programs were used in MD trajectories: gmxrms, gmxrmsf, gmx gyrate, gmxsasa, and gmxhbond. These MD analyses generated parameters values for root-mean-square deviation (RMSD), root-mean-square fluctuation (RMSF), radius of gyration, solvent accessible surface area (SASA), and number of h-bonds.

## Results

### Clinical manifestations

**FIGURE 1:**
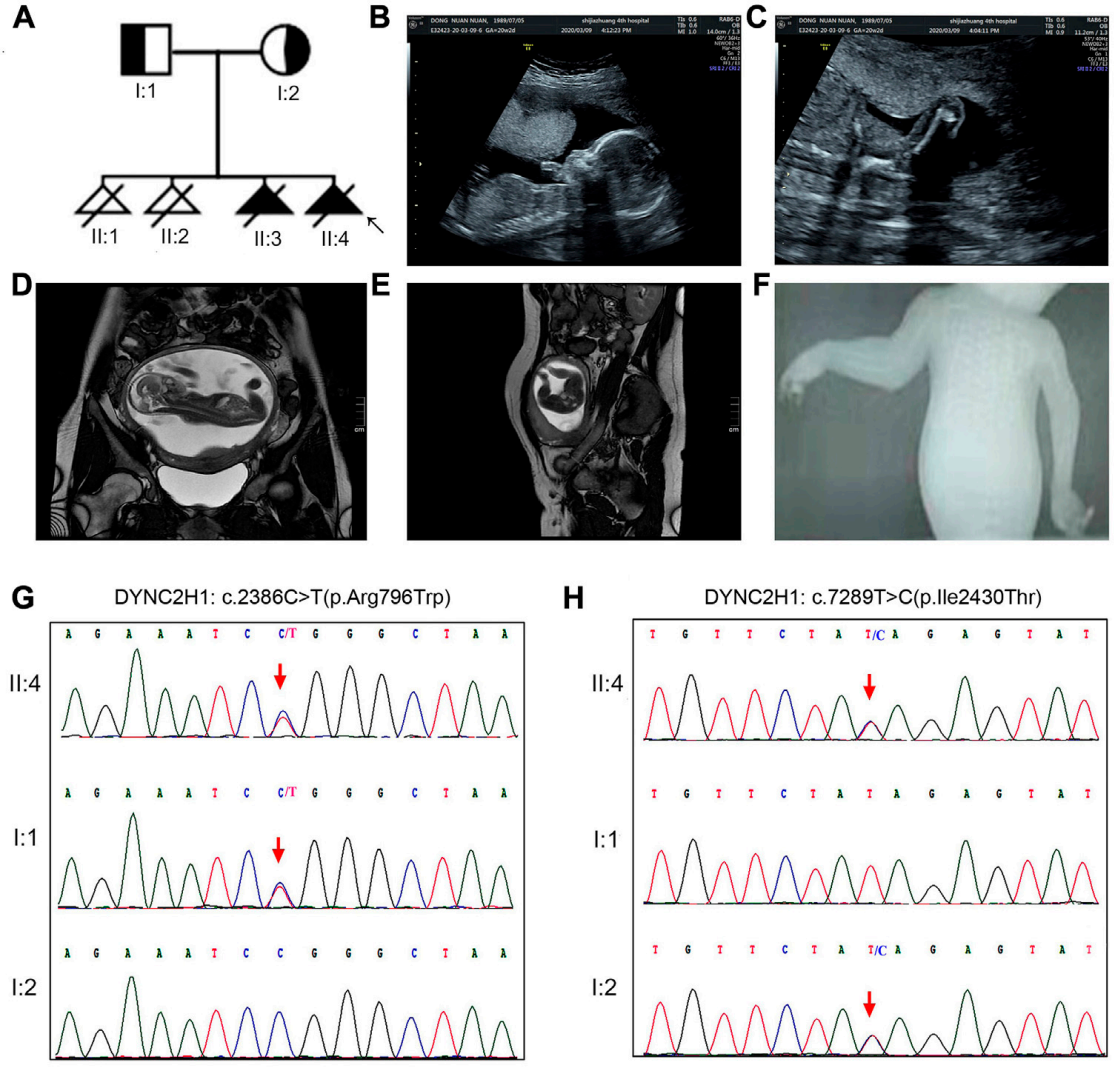
The clinical findings and genetic variation in case 1. **(A)** Pedigree diagram of the family with STRD3. **(B–E)** Ultrasonographic and MRI indications of the fetus in case 1: the fetus had extremely short limbs and a small, narrow thorax. **(F)** X-ray indications of the fetus in case 1: the fetus had a narrow thorax and short limbs, but no polydactyly. **(G, H)** The genetic variation identified in this case: proband 1 (II-4 in Case 1) carried two missense variants, namely c.2386C>T (p.Arg796Trp) and c.7289T>C (p.Ile2430Thr). Validation with Sanger sequencing demonstrated that the variants these probands carried were all inherited from their asymptomatic heterozygous carrier parents.

**Case 1.** A 32-year-old woman was referred to our center in March 2021 for previous multiple adverse gestations, when she was at the 17th^+^gestational week. Her husband was 31 years old, with no consanguineous relation to her. Based on the medical record and her personal dictation, we combed through the couple’s complete medical history and illustrated the pedigree in a diagram (Figure 1A). They had 3 previous pregnancies: in October 2016 and July 2017, they went through one spontaneous abortion and one stillbirth, respectively; in November 2018, the prenatal sonography revealed a 22-week fetus with short limbs and a narrow chest, which was diagnosed as thanatophoric dwarfism. That pregnancy was subsequently terminated.

In 2020, they had the 4th gestation, and the serological screening and NIPT (non-invasive prenatal testing) results were normal. At the 17th week, ultrasonic examination and MRI revealed that the fetus had extremely short limbs and small, bell-shaped chest, short ribs, rhizomelic shortening in all extremities (Figures 1B–E). Prenatal ultrasound did not identify abnormalities in the brain, heart, kidneys, or liver. No exposure history to tobacco smoke, alcohol, ir-radiation, or infectious diseases during the pregnancy were admitted. Pregnancy termination was performed at 22 gestational weeks with informed consent. Afterwards, X-ray imaging result demonstrated that the fetus had a narrow thorax and short limbs, but no polydactyly (Figure 1F). And whole-exome sequencing (WES) was introduced afterwards.

**FIGURE 2:**
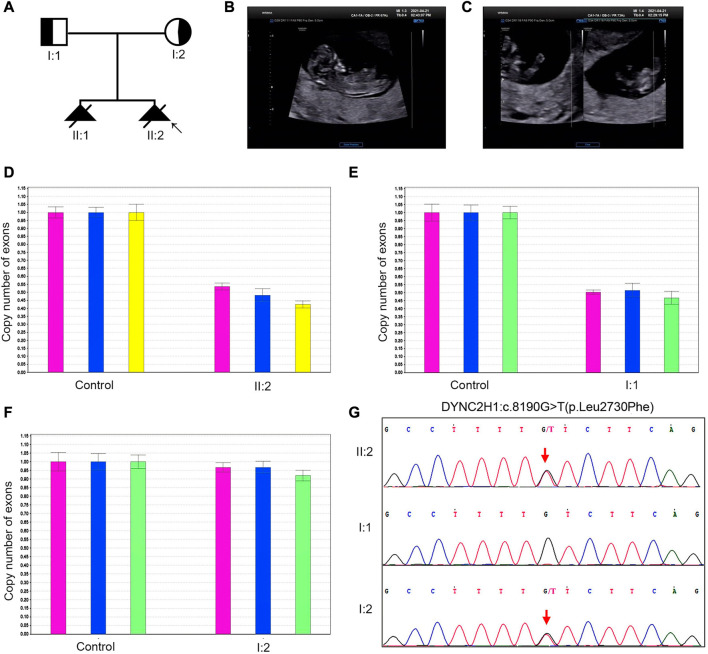
The clinical findings and genetic variation in case 2. **(A)** Pedigree diagram of the family with STRD3. **(B, C)** Ultrasonographic indications of the fetus in case 2: the fetus had extremely short limbs and bowing of long bones. **(D–G)** The genetic variation identified in case 2: proband 2 (II-2 in Case 2) carried an exonic deletion and a missense variant: exon (64–83)del and c.8190G>T (p.Leu2730Phe). qPCR validation confirmed one copy loss of the 64-83exons in *DYNC2H1*of proband 2 **(D)**. Validation with Sanger sequencing and qPCR demonstrated that the variants these probands carried were all inherited from their asymptomatic heterozygous carrier parents.

**Case 2.** A 31-year-old pregnant woman was referred to our center in April 2021. Her husband and her were non-consanguineous. The pedigree diagram was depicted in Figure 2A: in November 2018, a fetus was diagnosed with constricted thoracic cage, extremely shortened tubular bones and bowing of long bones, and then aborted at 12th week; in April 2021, ultrasonography revealed that their second fetus had extremely short limbs at the 13th week (Figures 2B, C). No brain, heart, kidney, or liver abnormality was found by prenatal ultrasonography. The woman denied to be exposed to tobacco smoke, alcohol, radiation or infectious diseases during pregnancy. After informed consent by the couple, genetic analysis including WES was also performed after induction.

### Genetic variations

**FIGURE 3:**
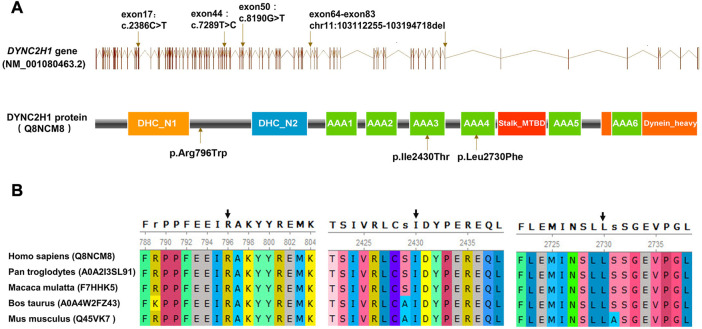
**(A)** Schematic diagram of the DYNC2H1 protein and the locations of the variants detected. **(B)** Conservation analysis of DYNC2H1 indicated that the protein at position 796, 2430 and 2730 are highly conserved in various species.

Karyotyping by G-bandeding showed that the results of the fetuses in both Case1 and 2 were normal, and array-CGH analysis did not reveal any genomic abnormality associated with known microdeletion or microduplication syndromes, either. On the other hand, according to the WES results, the two fetuses were recognized as positive with compound heterozygous variation in the *DYNC2H1* gene. To be specific, Proband 1 (II-4 in Case 1) carried two heterozygous missense variants, namely c.2386C>T (p.Arg796Trp) and c.7289T>C (p.Ile2430Thr) (Figures 1G, H); while Proband 2 (II-2 in Case 2) carried an exonic deletion, exon (64–83)del, and a missense variant, c.8190G>T (p.Leu2730Phe) (Figure 2G). qPCR validation confirmed the one copy loss of 64–83 exons in *DYNC2H1* of Proband 2 (Figure 2D). Validation with Sanger sequencing and qPCR demonstrated that the variants these probands carried were inherited from their asymptomatic heterozygous carrier parents, respectively (Figures 1G, H; Figures 2D–G). The location of each variant was illuminated in the gene and peptide diagrammatic sketches (Figure 3A).

### Conservatism analysis of missense variants

In this study, three missense variants were detected, which were *DYNC2H1*: c.2386C>T (p.Arg796Trp), c.7289T>C (p.Ile2430Thr) and c.8190G>T (p.Leu2730Phe). We analyzed the evolutionary conservatism of AA residues they affected. Results indicated that all three AAs maintained highly conserved among species (Figure 3B).

### Structural analysis and molecular dynamics simulation

**FIGURE 4:**
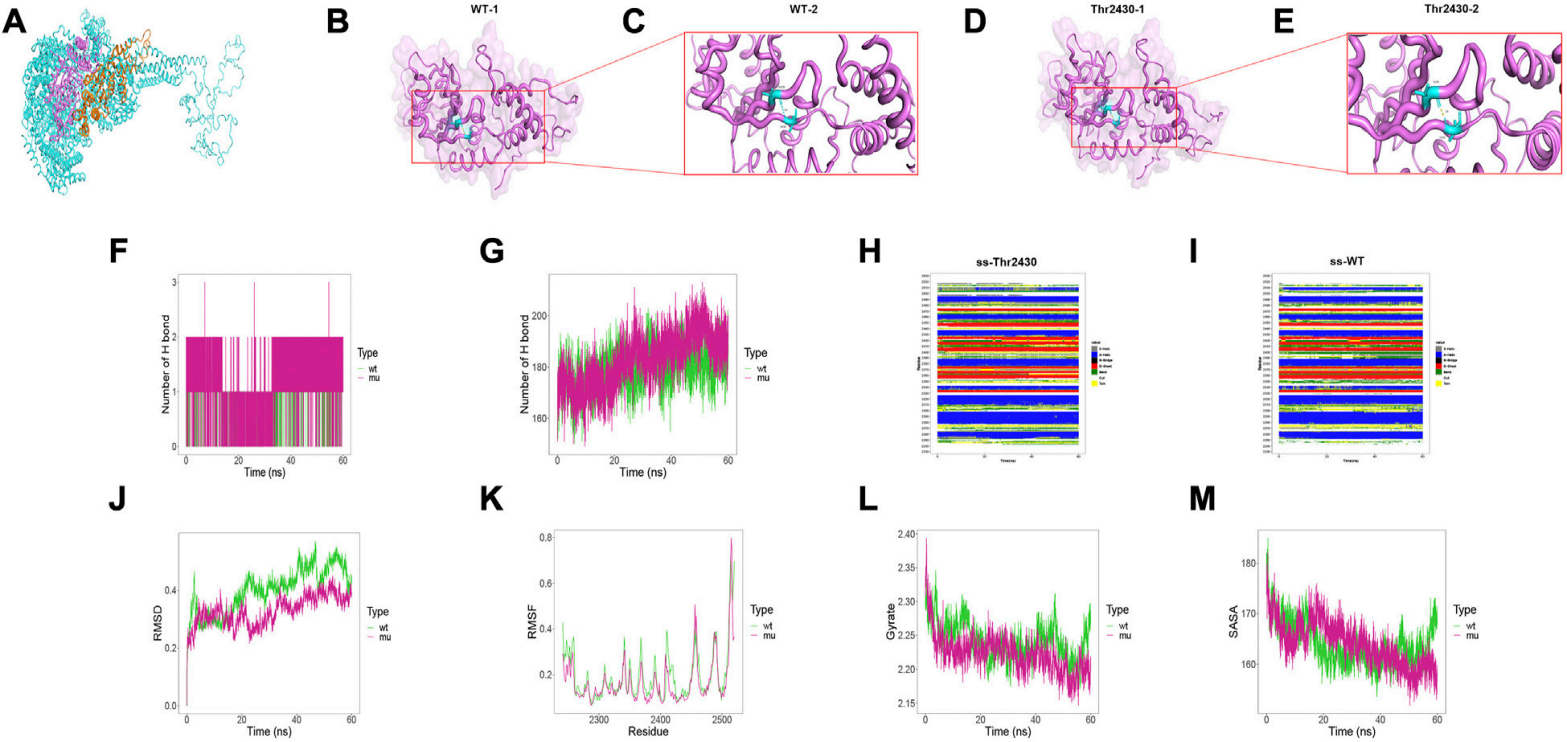
Results of structural analysis and molecular dynamics simulation of DYNC2H1: c.7289T>C (p.Ile2430Thr) variation. **(A)** Protein structure of DYNC2H1. **(B, C)** The wild-type structure of DYNC2H1: protein (DYNC2H1), and enlarged image of the segment containing Ile2430 residue. **(D, E)** The mutant structure of DYNC2H1, and segment containing the variant Thr2430 residue. **(F)** The number of hydrogen bonds formed between the target amino acid (meaning Ile2430 or Thr2430) and other residues. **(G)** Total number of hydrogen bonds in the wild-type model and the mutant model, respectively. **(H, I)** Comparison of local secondary structure data between Ile2430Thr mutant and wild-type. **(J)** RMSD: a numerical measurement indicating the difference between two structures. **(K)** RMSF: is a numerical measure similar to RMSD, but instead of indicating differences in position over time between entire structures; it calculates the flexibility of individual residues, or the extent to which a particular residue moves (fluctuates) during a simulation. **(L)** Gyrate: is a measure of the structural displacement of a protein atom with its common center of mass over the course of the simulation and provides comprehensive information about protein tightness over time. **(M)** SASA: measures the exposed surface in a protein structure accessible to solvent molecules.

To investigate the intramolecular effect of these missense variants, we performed structural analysis and MD simulation. Due to the structural inaccuracy of the region where p. Arg796Trp was located, we only analyzed the DYNC2H1 protein structures at 2241–2520 region (violet part in Figure 4A) and 2611–2880 region (orange part in Figure 4A), where p. Ile2430Thr and p. Leu2730Phe were located.

The results demonstrated that the Ile2430Thr variant affected the hydrogen bonding between amino acids inside the protein. Particularly, in the wild-type, Ile2430 formed hydrogen bonds with Val2290 residues, with the hydrogen bond length of 2.9Å; while the Ile2430Thr mutant formed a hydrogen bond with Val2290 with a hydrogen bond length of 1.9Å (Figures 4B–E; 4C and 4E are local amplifications of 4B and 4D, respectively). As for the MD results, first, the Thr2430 mutant formed more hydrogen bonds with other amino acids in the protein than the wild-type residue Ile2430 (Figure 4F); for the total number of hydrogen bonds inside the models within 60ns, the amount of Ile2430Thr mutant was more than that of the wild-type (Figure 4G). Besides, the Ile2430Thr variant resulted in a change in secondary structure around 2350th and 2400th residue (Figures 4H, I). Specifically, in wild-type, the secondary structure at 2350 position was dominated by BEND; yet in the mutant, the secondary structure alternated between BEND and TURN and was dominated by TURN. In wild-type, the secondary structure at 2400 position was dominated by BEND; yet in the mutant, the secondary structure alternated between BEND and TURN and was dominated by TURN. Finally, the Ile2430Thr variant resulted in decreased changes in protein structure (Figure 4J), decreased flexibility of amino acids in protein (Figure 4K), increased protein compactness (Figure 4L), and decreased surface area accessible to protein solvent (Figure 4M).

**FIGURE 5:**
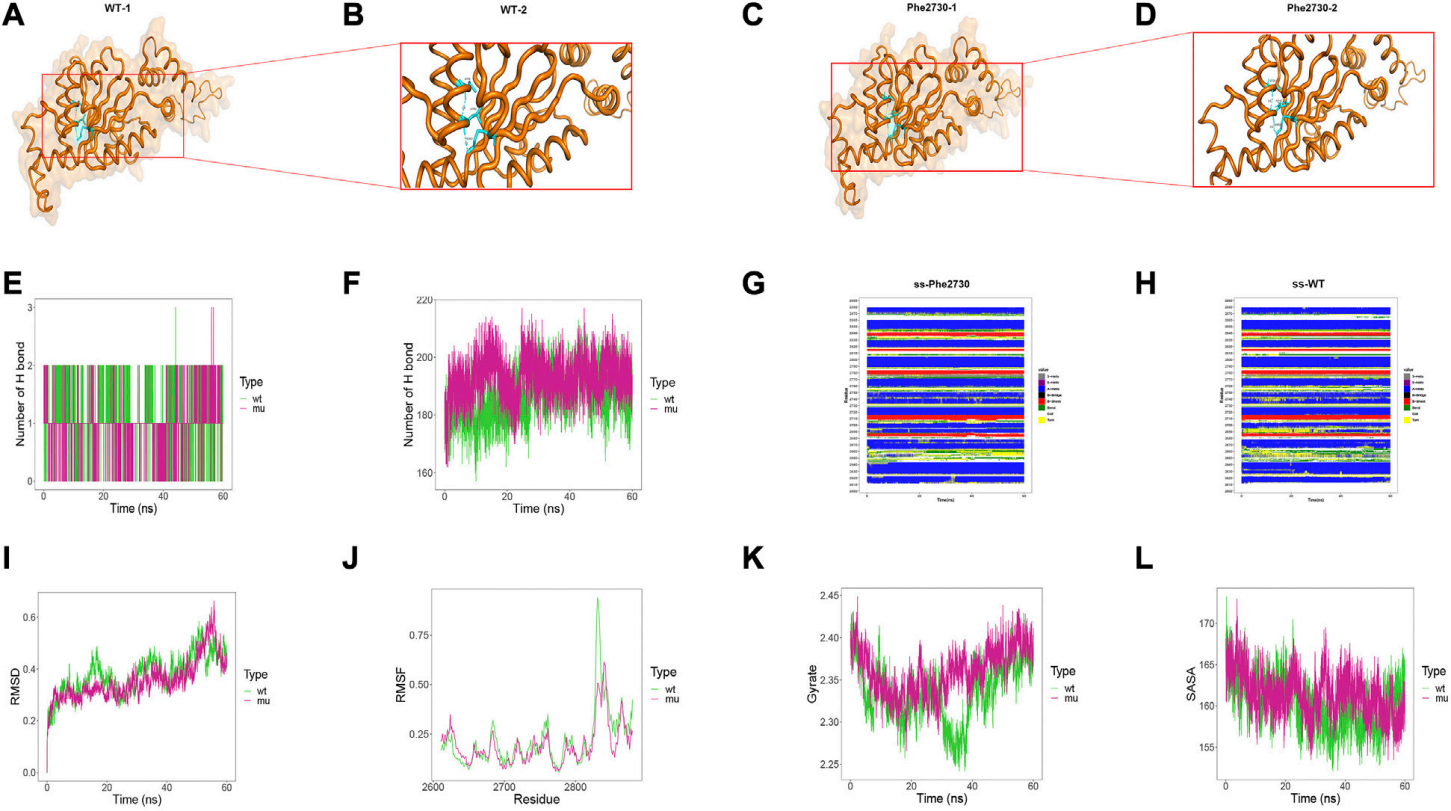
Results of structural analysis and molecular dynamics simulation of DYNC2H1: c.8190G>T (p.Leu2730Phe) variation. **(A, B)** The wild-type structure of DYNC2H1: protein (DYNC2H1), and enlarged image of the segment containing Leu2730 residue. **(C, D)** The mutant structure of DYNC2H1, and segment containing the variant Phe2730 residue. **(E)** The number of hydrogen bonds formed between the target amino acid (meaning Leu2730 or Phe2730) and other residues. **(F)** Total number of hydrogen bonds in the wild-type model and the mutant model, respectively. **(G, H)** Comparison of local secondary structure data between Leu2730Phe mutant and wild-type. **(I)** RMSD: a numerical measurement indicating the difference between two structures. **(J)** RMSF: is a numerical measure similar to RMSD, but instead of indicating differences in position over time between entire structures; it calculates the flexibility of individual residues, or the extent to which a particular residue moves (fluctuates) during a simulation. **(K)** Gyrate: is a measure of the structural displacement of a protein atom with its common center of mass over the course of the simulation and provides comprehensive information about protein tightness over time. **(L)** SASA: measures the exposed surface in a protein structure accessible to solvent molecules.

Structural result demonstrated that Leu2730Phe variant affected the hydrogen bonding between amino acids inside the protein. Particularly, Leu2730 and Phe2730 both formed hydrogen bonds with Ile2726 and Lys2802 residues, yet in the Leu2730Phe mutant the hydrogen bond length is shorter than that in the wild-type (Figures 5A–D; 5B and 5D are local amplifications of 5A and 5C, respectively). As for the MD results, there was no difference between Leu2730 and Phe2730 in the number of hydrogen bonds formed with other amino acids in the protein (Figure 5E); for the number of total hydrogen bonds inside the models within 60ns, the amount of Leu2730Phe mutant was more than that of the wild-type (Figure 5F). In wild-type, the secondary structure at 2690 position was dominated by H-helix; yet in the mutant, the secondary structure showed a high rate of BEND and TURN (Figures 5G, H). The Leu2730Phe mutation has little influence on RMSD, RMSF, Gyrate, and SASA (Figures 5I–L).

## Discussion

SRTD3 refers to a sort of autosomal recessive skeletal condition characterized by shortened limbs, narrow thorax, with or without polydactyly, non-skeletal involvement can include cleft lip/palate as well as anomalies of major organs such as the brain, eye, heart, kidneys, liver, pancreas, intestines, and genitalia (Chen et al., 2018). The prenatal ultrasound features of SRTD3 are similar to other skeletal diseases, so it is difficult to establish a definite diagnosis, which proposes a challenge in prenatal diagnosis and management on fetuses with similar early manifestations.

In this study, two families with experiences of multiple adverse gestations including recurrent fetal skeletal dysplasia were enrolled. Ultrasonography detection of the two fetuses revealed skeletal abnormalities characteristics of SRTD3, including extreme shortness of the limbs, and narrow thorax. Signs of polydactyly and non-skeletal symptoms were not noted in the two cases. In Case 1, WES detected a compound heterozygous variation in the *DYNC2H1* gene with two variants, c.2386C>T and c.7289T>C. The allele frequencies of c.2386C>T (p.Arg796Trp) and c.7289T>C (p.Ile2430Thr) in gnomAD database were 2.3822e-05 and 9.39391e-06 respectively. In agreement with autosomal-recessive segregation, the parents were heterozygous for these two variants, respectively: the father carried the heterozygous c.2386C>T variant while the mother carried the c.7289T>C variant. The c.2386C>T variant caused a replacement of Arg796 residue by a Trp amino acid, and c.7289T>C caused a substitution of Ile2430 residue by a Thr amino acid. According to the variant interpretation criteria by ACMG, c.2386C>T (p.Arg796Trp), and c.7289T>C (p.Ile2430Thr) variants were classified as VUS, with the evidence of PM2+PP3. The c.2386C>T (p.Arg796Trp) and c.7289T>C (p.Ile2430Thr) variants were predicted to be pathogenic by the SIFT algorithm, Mutation Taster, and PolyPhen-2.

In Case 2, a compound heterozygous variation in *DYNC2H1* gene with 2 variants, exon (64–83) del and c.8190G>T, was identified and confirmed. The former one was a novel variant not indexed in the databases of 1000G (https://www.internationalgenome.org/), gnomAD (http://gnomad.broadinstitute.org/), ExAC_EAS (http://exac.broadinstitute.org) and Berry Genomics inhouse database, which expanded the variation spectrum of *DYNC2H1* gene. The allele frequencies of c.8190G>T (p.Leu2730Phe) in the databases of EXAC and gnomAD were 2.7e-05 and 1.6 e-05 respectively. Consistent with autosomal-recessive segregation, the parents were heterozygous for the identified mutations: the father carried exon (64–83)del, while the mother carried c.8190G>T. According to the variant interpretation criteria by ACMG, the exon (64–83)del variant was classified as pathogenic, with the evidence of PVS1+PM2. According to the variant interpretation criteria by ACMG, the c.8190G>T (p.Leu2730Phe) variant was classified as VUS, with the evidence of PM2+PP3. The c.8190G>T variant caused a replacement of Leu2730 residue by a Phe amino acid, and this variant was predicted to be pathogenic by the SIFT algorithm, Mutation Taster, and PolyPhen-2. The three residues affected by c.2386C>T (p.Arg796Trp), c.7289T>C (p.Ile2430Thr) and c.8190G>T (p.Leu2730Phe) variants maintained conserved across species, which strongly supports the pathogenicity of these variants. The pathogenicity and ACMG classification of all the variants identified in DYNC2H1 were shown in Table 2.

**TABLE 2:** DYNC2H1 variants (cited from HGMD† database, in order of mutation position).

No.	Genomic coordinates	References base(s)	Variant base(s)	HGVS† description	ACMG
1	chr11:102980496–102980496	A	C	193A>C	VUS
2	chr11: 102980498–102980498	G	T	195G>T	Likely Pathogenic
3	chr11: 102984382–102984383	TA		313_314delAT	Likely Pathogenic
4	chr11:102984397–102984397	C	G	327C>G	Pathogenic
5	chr11:102984407–102984407	C	T	337C>T	Likely Pathogenic
6	chr11: 102985918–102985918	C	A	515C>A	VUS
7	chr11: 102985938–102985938	T	G	535T>G	VUS
8	chr11:102985939–102985939	G	A	536G>A	Pathogenic
9	chr11: 102987300–102987302	AGT	AAA	624_625delGTinsAA	Pathogenic
10	chr11: 102987302–102987302	T	A	625T>A	Pathogenic
11	chr11:102987417–102987417	G	A	740G>A	VUS
12	chr11:102988358–102988358	A	G	767-2A>G	Pathogenic
13	chr11:102988465–102988465	G	T	872G>T	VUS
14	chr11:102988533–102988533	T	C	940T>C	VUS
15	chr11:102988581–102988581	C	T	988C>T	Likely Pathogenic
16	chr11:102991188–102991188	A	G	1012A>G	VUS
17	chr11:102991254–102991254	C	T	1078C>T	Pathogenic
18	chr11:102991434–102991434	C	T	1151C>T	Likely Pathogenic
19	chr11:102991693–102991693	C	T	1288C>T	VUS
20	chr11:102991694–102991694	G	A	1289G>A	VUS
21	chr11:102991711–102991711	G	T	1306G>T	Pathogenic
22	chr11:102991767–102991767	T		1360+2delT	Pathogenic
23	chr11:102992161–102992161	T	G	1421T>G	VUS
24	chr11:102992166–102992166	G		1427delG	Likely Pathogenic
25	chr11:102992224–102992224	A	G	1484A>G	VUS
26	chr11:102993672–102993672	T	C	1604T>C	VUS
27	chr11:102993723–102993726	GTTT		1657_1660delTTGT	Pathogenic
28	chr11:102995926–102995926	C	T	1759C>T	Likely Pathogenic
29	chr11:102996011–102996016	TTATTC		1847_1852delTTCTTA	Likely Pathogenic
30	chr11:102996022–102996022	C	T	1855C>T	Pathogenic
31	chr11:102999730–102999730	T	A	1949T>A	Likely Pathogenic
32	chr11:102999734–102999734	G	A	1953G>A	Likely Pathogenic
33	chr11:103004369–103004369		T	2040dupT	Pathogenic
34	chr11:103004417–103004417	A	C	2087A>C	VUS
35	chr11:103005113–103005113	G	A	2170G>A	VUS
36	chr11:103006243–103006243	T	G	2225T>G	Likely Pathogenic
37	chr11:103006359–103006359	T	G	2341T>G	Likely Pathogenic
38	chr11:103006444–103006444	T	G	2346-5T>G	Likely Pathogenic
39	chr11:103006488–103006488	C		2386delC	Pathogenic
40	chr11:103006488–103006488	G	A	2386C>T	VUS
41	chr11:103006497–103006497	A	G	2394A>G	VUS
42	chr11:103006498–103006500	TAT		2398_2400delTAT	VUS
43	chr11:103006546–103006546	G	C	2443G>C	VUS
44	chr11:103006652–103006652	T	A	2549T>A	Likely Pathogenic
45	chr11:103006678–103006678	G	A	2574 + 1G>A	Pathogenic
46	chr11:103013996–103013996	G	A	2575-1G>A	Likely Pathogenic
47	chr11:103014034–103014034	T	C	2612T>C	VUS
48	chr11:103018547–103018549	ACT	AG	2750_2751delCTinsG	Likely Pathogenic
49	chr11:103018617–103018617	G	C	2818 + 1G>C	Pathogenic
50	chr11:103019205–103019205	A	G	2819-14A>G	VUS
51	chr11:103022910–103022910	C	T	2992C>T	Likely Pathogenic
52	chr11:103022977–103022977	T	G	3059T>G	Pathogenic
53	chr11:103023013–103023013	A		3095delA	Pathogenic
54	chr11:103024028–103024028	A	G	3097-4A>G	Benign
55	chr11:103024068–103024068	C	T	3133C>T	Likely Pathogenic
56	chr11:103024187–103024187	A	C	3252A>C	VUS
57	chr11:103024190–103024190		A	3262dupA	Pathogenic
58	chr11:103025179–103025179	G	A	3303-1G>A	Likely Pathogenic
59	chr11:103025208–103025208	G	A	3331G>A	VUS
60	chr11:103025230–103025230	G		3353delG	Pathogenic
61	chr11:103025335–103025335	G	A	3458G>A	VUS
62	chr11:103025423–103025423	G	A	3459-1G>A	Pathogenic
63	chr11:103026124–103026124	T	G	3638T>G	VUS
64	chr11:103026168–103026168	C	A	3682C>A	VUS
65	chr11:103026180–103026180	G	A	3694G>A	VUS
66	chr11:103026205–103026205	T	C	3719T>C	VUS
67	chr11:103027219–103027219	G	C	3847G>C	VUS
68	chr11:103027444–103027444	C	T	4072C>T	VUS
69	chr11:103027445–103027445	G	A	4073G>A	Likely Pathogenic
70	chr11:103029413–103029413	A	G	4135A>G	VUS
71	chr11:103029438–103029438		AGTCACAAC	4162_4170dupGTCACAACA	Likely Pathogenic
72	chr11:103029637–103029637	A	G	4261-2A>G	Likely Pathogenic
73	chr11:103029645–103029645	C	T	4267C>T	Pathogenic
74	chr11:103029703–103029703	G	A	4325G>A	VUS
75	chr11:103029729–103029729	C	T	4351C>T	Likely Pathogenic
76	chr11:103031700–103031700	T	C	4418T>C	Likely Pathogenic
77	chr11:103031737–103031737	T		4458delT	Pathogenic
78	chr11:103031764–103031768	TAATG		4484_4488delATGTA	Likely Pathogenic
79	chr11:103033818–103033818	G	A	4553G>A	Likely Pathogenic
80	chr11:103033875–103033875	A	G	4610A>G	VUS
81	chr11:103036640–103036640	C	T	4625C>T	Likely Pathogenic
82	chr11:103036714–103036714	C	G	4699C>G	Likely Pathogenic
83	chr11:103039541–103039541	T	A	4820T>A	Likely Pathogenic
84	chr11:103039630–103039630	G	T	4909G>T	VUS
85	chr11:103039685–103039685	A	G	4964A>G	Likely Pathogenic
86	chr11:103040955–103040955	C	T	5087C>T	Likely Pathogenic
87	chr11:103040997–103040997	T	A	5129T>A	Likely Pathogenic
88	chr11:103041020–103041020	G	T	5151 + 1G>T	Likely Pathogenic
89	chr11:103041639–103041639	C	T	5176C>T	Pathogenic
90	chr11:103041680–103041680	T		5220delT	Likely Pathogenic
91	chr11:103043839–103043839	T	G	5363T>G	VUS
92	chr11:103043994–103043994		TA	5520_5521dupAT	Likely Pathogenic
93	chr11:103044036–103044036	T	C	5558 + 2T>C	Pathogenic
94	chr11:103044837–103044837	C		5612delC	Pathogenic
95	chr11:103046970–103046971	AA		5682_5683delAA	Pathogenic
96	chr11:103047080–103047080	T	A	5791T>A	VUS
97	chr11:103047082–103047082	G	C	5793G>C	VUS
98	chr11:103048286–103048286	T	A	5876T>A	Likely Pathogenic
99	chr11:103048330–103048330	G	T	5920G>T	Pathogenic
100	chr11:103048334–103048334	T		5925delT	Pathogenic
101	chr11:103048369–103048369	A	G	5959A>G	Likely Pathogenic
102	chr11:103048381–103048381	A	T	5971A>T	Pathogenic
103	chr11:103048382–103048382	T	A	5972T>A	Likely Pathogenic
104	chr11:103048393–103048393	G	A	5983G>A	Pathogenic
105	chr11:103048394–103048394	C	T	5984C>T	Likely Pathogenic
106	chr11:103048445–103048445	C	T	6035C>T	VUS
107	chr11:103048453–103048453	C	T	6043C>T	Likely Pathogenic
108	chr11:103048454–103048454	G	A	6044G>A	VUS
109	chr11:103048457–103048457	A	G	6047A>G	Likely Pathogenic
110	chr11:103048526–103048526	G	A	6116G>A	Likely Pathogenic
111	chr11:103049776–103049776	G	C	6161G>C	Likely Pathogenic
112	chr11:103049821–103049821	T	C	6206T>C	VUS
113	chr11:103049880–103049880	A	G	6265A>G	Likely Pathogenic
114	chr11:103049886–103049886	A	G	6271A>G	VUS
115	chr11:103049959–103049959	T	A	6344T>A	VUS
116	chr11:103052525–103052525	G	T	6387G>T	VUS
117	chr11:103055609–103055609	G	A	6478-16G>A	VUS
118	chr11:103055627–103055627	T	A	6480T>A	Likely Pathogenic
119	chr11:103055692–103055692	G	A	6545G>A	Likely Pathogenic
120	chr11:103055698–103055698	A	T	6551A>T	Likely Benign
121	chr11:103055709–103055709	T	C	6562T>C	Likely Pathogenic
122	chr11:103055721–103055721	C	T	6574C>T	Likely Pathogenic
123	chr11:103055735–103055737	TGG		6591_6593delTGG	VUS
124	chr11:103055761–103055761	G	A	6614G>A	Pathogenic
125	chr11:103055779–103055779	A	T	6632A>T	VUS
126	chr11:103056969–103056969	A	G	6634-2A>G	Pathogenic
127	chr11:103057016–103057016	A	G	6679A>G	VUS
128	chr11:103057146–103057146	G	A	6809G>A	VUS
129	chr11:103057171–103057171	G	T	6834G>T	VUS
130	chr11:103057194–103057194	C	T	6857C>T	VUS
131	chr11:103057203–103057203	T	C	6866T>C	Likely Pathogenic
132	chr11:103057220–103057220	T	C	6883T>C	Likely Pathogenic
133	chr11:103058085–103058085	G	A	6910G>A	Likely Pathogenic
134	chr11:103058253–103058253	G	T	7078G>T	VUS
135	chr11:103058260–103058260	A	G	7085A>G	Likely Pathogenic
136	chr11:103058287–103058287	C	T	7112C>T	Likely Pathogenic
137	chr11:103058304–103058304	T	G	7129T>G	VUS
138	chr11:103059233–103059233	C	T	7148C>T	VUS
139	chr11:103059353–103059353	C	A	7268C>A	Likely Pathogenic
140	chr11:103059361–103059361	C	T	7276C>T	VUS
141	chr11:103059362–103059362	G	T	7277G>T	Likely Pathogenic
142	chr11:103059374–103059374	A	G	7289T>C	VUS
143	chr11:103060490–103060490	G	T	7382G>T	VUS
144	chr11:103060517–103060517	C	G	7409C>G	Likely Pathogenic
145	chr11:103060548–103060548	A	G	7437 + 3A>G	VUS
146	chr11:103062244–103062244	A	G	7438-2A>G	Pathogenic
147	chr11:1103062249–103062249	C	T	7441C>T	Pathogenic
148	chr11:103062250–103062250	G	A	7442G>A	VUS
149	chr11:103062294–103062294	C	T	7486C>T	VUS
150	chr11:103062333–103062333	T	C	7525T>C	Likely Pathogenic
151	chr11:103062347–103062347	A	T	7539A>T	VUS
152	chr11:103062846–103062846	G	C	7561G>C	VUS
153	chr11:103062862–103062862	T	G	7577T>G	Likely Pathogenic
154	chr11:103062879–103062879	C	T	7594C>T	Likely Pathogenic
155	chr11:103062928–103062928	T	C	7643T>C	Likely Pathogenic
156	chr11:103062948–103062948	G	A	7663G>A	Likely Pathogenic
157	chr11:103068671–103068671	A	G	7718A>G	VUS
158	chr11:103068724–103068732	CCATTACCT		7774_7782delTTACCTCCA	Likely Pathogenic
159	chr11:103068737–103068737	A	G	7784A>G	Likely Benign
160	chr11:103070000–103070000	T	C	7883T>C	VUS
161	chr11:103070036–103070036	T	C	7919T>C	VUS
162	chr11:103070042–103070042	G	C	7925G>C	VUS
163	chr11:103070062–103070062	G	T	7945G>T	Likely Pathogenic
164	chr11:103070083–103070083	C	T	7966C>T	Likely Pathogenic
165	chr11:103070084–103070084	G	A	7967G>A	VUS
166	chr11:103070084–103070084	G	T	7967G>T	Likely Pathogenic
167	chr11:103070098–103070098	C	T	7981C>T	VUS
168	chr11:103070101–103070101	C	T	7984C>T	Pathogenic
169	chr11:103070102–103070102	G	A	7985G>A	Likely Pathogenic
170	chr11:103070104–103070104	A	C	7987A>C	VUS
171	chr11:103070129–103070129	T	C	8012T>C	Likely Pathogenic
172	chr11:103070167–103070167	G	T	8050G>T	Pathogenic
173	chr11:103070179–103070179	A		8063delA	Likely Pathogenic
174	chr11:103070187–103070187	C	G	8070C>G	Likely Pathogenic
175	chr11:103070194–103070194	G	T	8077G>T	VUS
176	chr11:103070831–103070831	C	A	8145C>A	Pathogenic
177	chr11:103070876–103070876-	C	A	8190G>T	VUS
178	chr11:103070883–103070883	G	T	8197G>T	Likely Pathogenic
179	chr11:103074471–103074471	T		8283delT	Pathogenic
180	chr11:103074506–103074506	G	A	8311 + 1G>A	Pathogenic
181	chr11:103075552–103075552	A	T	8313A>T	VUS
182	chr11:103075578–103075578	T	C	8339T>C	VUS
183	chr11:103075628–103075636	CCAGCTTTG		8390_8398delCAGCTTTGC	VUS
184	chr11:103075673–103075673	T		8434delT	Pathogenic
185	chr11:103080607–103080607	A	G	8457A>G	VUS
186	chr11:103080662–103080662	C	T	8512C>T	Pathogenic
187	chr11:103080677–103080677	A		8534delA	Pathogenic
188	chr11:103082568–103082568	G		8590delG	Pathogenic
189	chr11:103082595–103082595	A	G	8617A>G	VUS
190	chr11:103082669–103082669	G	A	8691G>A	VUS
191	chr11:103086484–103086484	T	C	8729T>C	Likely Pathogenic
192	chr11:103086520–103086521	GT		8769_8770delGT	Pathogenic
193	chr11:103091415–103091415	C	T	9010C>T	VUS
194	chr11:103091449–103091449	A	G	9044A>G	Likely Pathogenic
195	chr11:103091450–103091450	T	G	9045T>G	Likely Pathogenic
196	chr11:103093704–103093704	G	A	9242G>A	VUS
197	chr11:103093816–103093816	G	A	9353 + 1G>A	Pathogenic
198	chr11:103104887–103104887	C	T	9565C>T	Pathogenic
199	chr11:103106471–103106471	A	G	9638A>G	VUS
200	chr11:103106494–103106494	C	T	9661C>T	VUS
201	chr11:103107157–103107157	A	G	9710-2A>G	Pathogenic
202	chr11:103107208–103107212	GAAAA		9760_9764delAAAAG	Pathogenic
203	chr11:103107263–103107263	T	A	9814T>A	Likely Benign
204	chr11:103107266–103107266	C	T	9817C>T	Pathogenic
205	chr11:103112255–103194718			EX64-EX83 Del	Likely Pathogenic
206	chr11:103112272–103112272	C	G	9836C>G	Pathogenic
207	chr11:103114423–103114423	G	A	9842G>A	VUS
208	chr11:103114425–103114425	C	T	9844C>T	Pathogenic
209	chr11:103114446–103114446	G	A	9865G>A	Likely Pathogenic
210	chr11:103114510–103114510	T	C	9929T>C	VUS
211	chr11:103116017–103116017	G		9977delG	Likely Pathogenic
212	chr11:103116062–103116062	C	G	10022C>G	VUS
213	chr11:103116085–103116085	C	T	10045C>T	Pathogenic
214	chr11:103116103–103116103	G	T	10063G>T	Pathogenic
215	chr11:103116105–103116105	T	G	10063 + 2T>G	Pathogenic
216	chr11:103124070–103124070	C	T	10120C>T	Likely Pathogenic
217	chr11:103124071–103124071	G	A	10121G>A	Likely Pathogenic
218	chr11:103124076–103124076	T	C	10126T>C	Likely Pathogenic
219	chr11:103124076–103124076	T		10130delT	Pathogenic
220	chr11:103124113–103124113	C	T	10163C>T	Likely Pathogenic
221	chr11:103124169–103124169	C	T	10219C>T	Pathogenic
222	chr11:103126224–103126224	G	A	10308G>A	VUS
223	chr11:103126259–103126259	T	C	10343T>C	Pathogenic
224	chr11:103128446–103128446	T	C	10592T>C	VUS
225	chr11:103128448–103128448	C	T	10594C>T	Pathogenic
226	chr11:103128460–103128460	C	T	10606C>T	Pathogenic
227	chr11:103128464–103128464	C	T	10610C>T	VUS
228	chr11:103128478–103128478	C	T	10624C>T	Pathogenic
229	chr11:103130659–103130659	T	C	10669T>C	VUS
230	chr11:103130699–103130702	TATT		10711_10714delTTTA	Likely Pathogenic
231	chr11:103151092–103151092	T	A	10732T>A	VUS
232	chr11:103153788–103153788	C	T	10885C>T	Likely Pathogenic
233	chr11:103153789–103153789	G	C	10886G>C	VUS
234	chr11:103156993–103156993	C	T	10921C>T	Likely Pathogenic
235	chr11:103157009–103157009	C		10939delC	Pathogenic
236	chr11:103173926–103173926	T	C	11221T>C	VUS
237	chr11:103173935–103173935	C	T	11230C>T	VUS
238	chr11:103173983–103173983	G	A	11277 + 1G>A	Likely Pathogenic
239	chr11:103175330–103175330	A	G	11284A>G	VUS
240	chr11:103175337–103175337	A	G	11291A>G	Likely Pathogenic
241	chr11:103175379–103175379	C	T	11333C>T	Likely Pathogenic
242	chr11:103178483–103178483	C	T	11437C>T	VUS
243	chr11:103178484–103178484	G	A	11438G>A	VUS
244	chr11:103178533–103178534	AC		11488_11489delCA	Likely Pathogenic
245	chr11:103178538–103178538	C	A	11492C>A	Likely Pathogenic
246	chr11:103182652–103182652	T	G	11560T>G	VUS
247	chr11:103182710–103182710-	G		11618delG	Pathogenic
248	chr11:103187334–103187337	GACA		11734_11737delAGAC	Pathogenic
249	chr11:103187341–103187342	TT		11741_11742delTT	Likely Pathogenic
250	chr11103191758-103191758	G	A	11747G>A	VUS
251	chr11:103191861–103191861	C	G	11850C>G	Pathogenic
252	chr11:103270549–103270549	T	G	12336T>G	VUS
253	chr11:103306683–103306683	T	C	12400T>C	VUS
254	chr11:103306714–103306714	C	G	12431C>G	Likely Pathogenic
255	chr11:103306743–103306743	C	T	12460C>T	Likely Pathogenic
256	chr11:103325912–103325912	A	G	12478-2A>G	Likely Pathogenic
257	chr11:103325921–103325924	TAGA		12487_12490delGATA	Pathogenic
258	chr11:103325942–103325942	C	G	12506C>G	VUS
259	chr11:103325974–103325974	C		12538delC	Likely Pathogenic
260	chr11:103326007–103326007	C	A	12571C>A	VUS
261	chr11:103326024–103326024	G	T	12587 + 1G>T	Likely Pathogenic
262	chr11:103327017–103327017		TG	12605_12606dupTG	Pathogenic
263	chr11:103327078–103327078		T	12663_12664insT	Pathogenic
264	chr11:103339363–103339363	T	G	12716T>G	VUS
265	chr11:103339395–103339395	T	A	12748T>A	VUS
266	chr11:103349863–103349863	T	C	12827T>C	Likely Pathogenic
267	chr11:103349886–103349886	A	G	12850A>G	Pathogenic
268	chr11:103349893–103349893	G	C	12857G>C	VUS
269	chr11:103349953–103349953	G	A	12917G>A	VUS

† HGMD, The Human Gene Mutation Database (http://www.hgmd.cf.ac.uk/ac/index.php); HGVS, Human Genome Variation Society (http://www.hgvs.org/).

The DYNC2H1 protein consists of an N-terminal tail (DHC_N1), a linker domain (DHC_N2), six identifiable AAA-ATPase domains, a stalk between AAA domains 4 and 5 in the microtubule binding domain (stalk MTBD), and a conserved C-terminal domain arranged on top of the ATPase ring (Carter et al., 2011). DYNC2H1 is essential for ciliogenesis and plays an important role in Hedgehog signaling events which are critical to human skeletal development (Krakow et al., 2000; Pazour et al., 2006). *DYNC2H1* encodes a subunit of cytoplasmic dynein complex, a component of IFTA involved in the retrograde transport from the ciliary tip to the basal body of the ciliary axoneme and plays a role in the generation and maintenance of mammalian cilia (Baujat et al., 2013). Variants in *DYNC2H1* have been associated with a heterogeneous spectrum of conditions related to altered primary cilium function that often involve polydactyly, abnormal skeletogenesis, and polycystic kidneys (Chen et al., 2016). Schmidts et al. (2013a) indicated that *DYNC2H1* missense mutations altered protein function, yet the effects might be “mild” or submorphic. Merrill et al. (2009) hypothesized that homozygosity for two null alleles would lead to early embryonic lethality, but a series of phenotypes with various severity could result from a combination of multiple missense and null mutations. The genotype-phenotype correlation of *DYNC2H1* is pending further elucidation along with larger genetic data.

The c.2386C>T variant affects the highly conserved arginine residues in the stem domain, while the c.7289T>C and the c.8190G>T missense variants are located within the ATP binding and hydrolysis domains (AAA3 and AAA4, respectively). These domains play an important regulatory role for ATP binding (Schmidts et al., 2013a). Blockage of ATP hydrolysis at AAA3 or AAA4 affects the catalytic and mechanical force production activities of dynein (Cho et al., 2008). Modeling the elimination of nucleotide binding at AAA2–4 domains cytoplasmic dynein indicated a severe slowed down in the microtubule sliding activity of the protein, implying dysfunctional motor activity (Zhang et al., 2018). The c.7289T>C and the c.8190G>T were missense changes affecting the ATP binding and hydrolysis domains of the protein (AAA3 and AAA4, respectively), which may disrupt the motor integrity, and interfering with proper retrograde IFT activity. According to the results of structural and MD analysis, the c.7289T>C (p.Ile2430Thr) variant was most likely to significantly affect both local and global hydrogen bond formation to alter protein stability, while also disrupting the desired secondary structure of the protein, thereby disrupting the binding of the protein to ATP. However, the c.8190G>T (p.Leu2730Phe) variant has less influence on the molecular dynamics. Further functional experiments are necessary, not only to clarify the effects of each variant on the protein itself, but also to understand the mechanistically contributes to the pathology seen in the skeletal ciliopathies.

For any future pregnancy of the couples in this study, the recurrent risk of SRTD3 condition would be 25%. Given such circumstances, the couples were informed of reproductive options such as prenatal testing and preimplantation genetic diagnosis (PGD). Proper genetic counseling for the affected family is essential in the case of rare genetic diseases. Furthermore, parenteral genetic screening/diagnosis is the best strategy for managing this disease, which currently has no therapy (Alyafee et al., 2021a; Alyafee et al., 2021b; Alyafee et al., 2022). Reporting additional cases associated with this gene would help identify genotype–phenotype correlations and lead to clinical trials in the future (Alfadhel et al., 2019).

In summary, this study detected two compound heterozygous variation in *DYNC2H1* including one novel deletion: exon (64–83)del. Our findings clarified the cause of fetal skeletal dysplasias in the subject families, provided guidance for their future pregnancies, and highlighted the value of WES in diagnosis of skeletal dysplasia with unclear prenatal indications.

## Data Availability

The datasets presented in this study can be found in online repositories. The sequencing results have been deposited to the Figshare repository and can be accessed via the following links: https://doi.org/10.6084/m9.figshare.21738650.v3 and https://doi.org/10.6084/m9.figshare.21738068.v4.
